# Characterization of *Brucella* spp. and other abortigenic pathogens from aborted tissues of cattle and goats in Rwanda

**DOI:** 10.1002/vms3.805

**Published:** 2022-04-14

**Authors:** Jean Bosco Ntivuguruzwa, Francis Babaman Kolo, Emil Ivan Mwikarago, Henriette van Heerden

**Affiliations:** ^1^ Department of Veterinary Tropical Diseases Faculty of Veterinary Science University of Pretoria Pretoria South Africa; ^2^ Department of Veterinary Medicine College of Veterinary Medicine University of Rwanda Kigali Rwanda; ^3^ Department of Human Medicine and Device assessment and Registration, Rwanda Food and Drug Administration Kigali Rwanda

**Keywords:** abortigenic pathogens, cattle, goats, characterization

## Abstract

**Background:**

Abortions cause tremendous economic losses in food‐producing animals and may lead to food insecurity.

**Objectives:**

This study aimed to characterize *Brucella* spp. and other abortigenic pathogens from aborted tissues of cattle.

**Methods:**

For cattle, aborted tissues (*n* = 19) were cultured, and *Brucella* spp. were detected using the genus‐specific 16S‐23S ribosomal DNA interspacer region (ITS) assay and speciated using *Brucella abortus*, *Brucella melitensis*, *Brucella ovis*, and *Brucella suis* (AMOS) and Bruce‐ladder PCR assays. *Brucella* negative samples were screened using the eight abortigenic pathogens PCR panel. Samples from an abortion outbreak that occurred within a goat tribe were included in this investigation. Sera of females (*n* = 8) and males (*n* = 2) were analyzed using the Rose Bengal Test (RBT) and indirect enzyme‐linked immunosorbent assay (i‐ELISA), while vaginal swabs (*n* = 3) and aborted tissues (*n* = 1) were cultured and characterized.

**Results:**

The ITS‐PCR detected *Brucella* DNA in cultures from two aborted tissues of cattle (10.5%, [2/19]), which were identified as *B. melitensis* (*n* = 1), and *B. abortus* (*n* = 1) using AMOS and Bruce‐ladder PCR assays. *Campylobacter fetus* (*n* = 7) and *Leptospira* spp. (*n* = 4) including co‐infections (*n* = 2) of *C. fetus* and *Leptospira* spp. were identified from the *Brucella* negative samples of cattle. Goats (100.0%, 10/10) were brucellosis seropositive on RBT and i‐ELISA. Mixed infections caused by *B. melitensis* and *B. abortus* were isolated from the vaginal swabs (*n* = 3) and aborted tissues (*n* = 1).

**Discussion and conclusions:**

This is the first identification of abortion‐associated pathogens in aborted cattle indicating the enormous financial losses and a threat to public health. It is therefore essential to include these identified pathogens in the surveillance scheme of veterinary and human services.

## Graphical abstract

This is the first identification of abortion‐associated pathogens (*B. abortus*, *B. melitensis*, *C. fetus*, and *Leptospira* spp.) in aborted cattle samples in Rwanda indicating the enormous financial losses to cattle owners and a threat to public health. It is therefore essential to include these identified pathogens in the surveillance scheme of veterinary and human services as well as raise its awareness among caretakers, abattoir workers, and laboratory personnel.

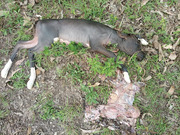

## 1 INTRODUCTION

Abortion is the premature expulsion of a dead fetus due to abnormalities of the reproductive tissues (Cabell, 2007; Samartino & Enright, 1993). Abortions cause tremendous economic losses in food‐producing animals and lead to food insecurity (Singh et al., 2015). Abortion is a clinical sign with multiple aetiologies including nutritional deficiencies and infectious pathogens (da Silva et al., 2009). Infectious pathogens account for 90.0% of ruminants’ abortions, and these pathogens include bacteria, viruses, protozoans, and fungi (da Silva et al., 2009). Among these pathogens, the genus *Brucella* is among the major causes of infectious abortions in ruminants (da Silva et al., 2009). Other abortigenic pathogens include *Campylobacter fetus*, and *Leptospira* spp. (da Silva et al., 2009).

*Brucella* spp. are contagious pathogens causing abortions in the last term of gestation (Samartino & Enright, 1993) of domestic and wildlife animals, as well as humans (Corbel, 2006). Zoonotic species of the genus *Brucella* were primarily isolated from aborting hosts. For instance, *Brucella melitensis* affects sheep and goats (Zammit, 1905), *Brucella abortus* affects cattle (Bang, 1897), *Brucella suis* affects pigs (Traum, 1914), and *Brucella canis* affects dogs (Kimberling et al., 1966). These species grow massively in the presence of erythritol, a normal constituent of amniotic fluid, leading to abortions in cows, ewes, does, and sow (Keppie et al., 1965; Pearce et al., 1962). During an abortion episode caused by *B. abortus*, aborted tissues contain more than 10^14^ bacteria, which is 10^5^ times the estimated infectious dose of heifers vaccinated with S19 (Corner et al., 1983).

The species *C. fetus* is a zoonotic pathogen of veterinary importance. It is divided into *C. fetus* subsp. *fetus* (Cff) and *C. fetus* subsp. *venerealis* (Cfv) (Véron & Chatelain, 1973). Cfv is a cattle‐restricted pathogen, which causes genital campylobacteriosis characterized by infertility, low conception rate, and abortions worldwide (Ishtifaq et al., 2020). Cff is a pathogen that cannot survive in the bovine intestine and causes reproductive disorders in sheep and cattle (Blaser et al., 2008). It is an opportunistic pathogen infecting mainly immune‐compromised patients (Tremblay et al., 2003). *Leptospira* spp. are zoonotic pathogenic spirochaetes of the genus *Leptospira* that cause abortion, stillbirth fetuses, decreased milk production, and low fertility (Momtaz & Moshkelani, 2012). Although these pathogens have never been reported in livestock in Rwanda, they are of considerable financial and economic losses resulting from reproductive failure.

Various reproductive disorders that have been reported in the cattle industry include higher incidences of abortions, retained placenta, infertility, and longer calving intervals (Chatikobo et al., 2009). Although a history of abortion was a significant predictor of brucellosis (Ndazigaruye et al., 2018; Ntivuguruzwa et al., 2020), there are several unreported cases of abortions in *Brucella* seronegative cattle. Furthermore, *Brucella* spp. or other abortifacient pathogens have never been detected from aborted tissues of cattle. Therefore, this study investigated the presence of *Brucella* spp. and other eight abortigenic pathogens from aborted tissues of cattle from January 2018 to October 2019.

## 2 MATERIALS AND METHODS

### 2.1 Study design and sample size

**FIGURE 1:**
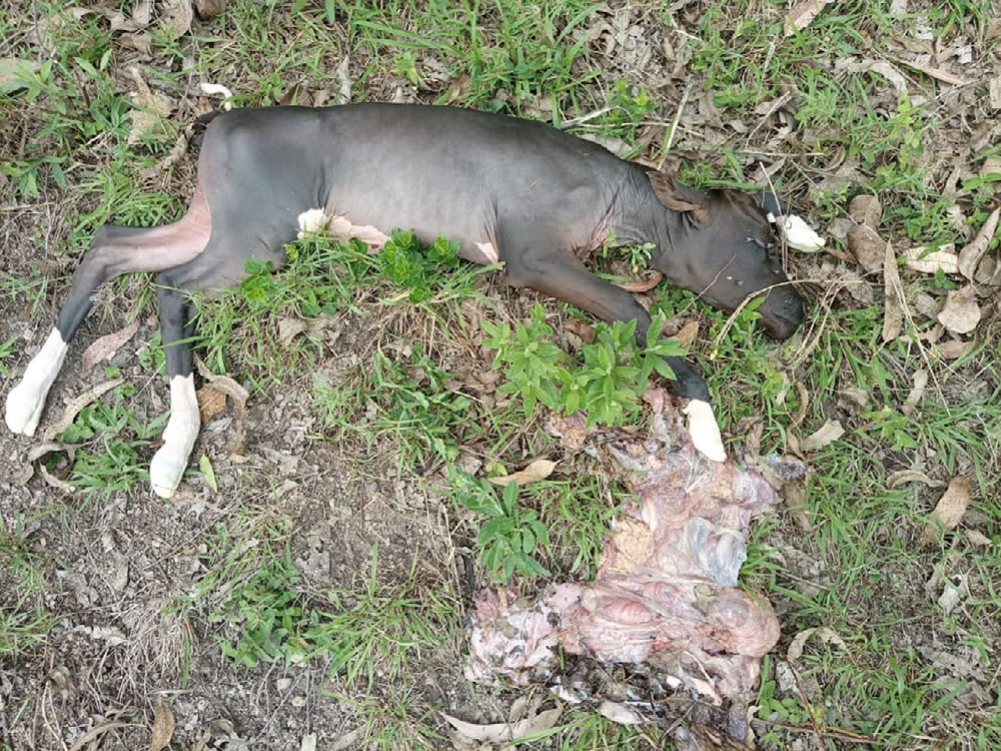
Abortion case which occurred in cattle from Nyanza district in November 2019

**FIGURE 2:**
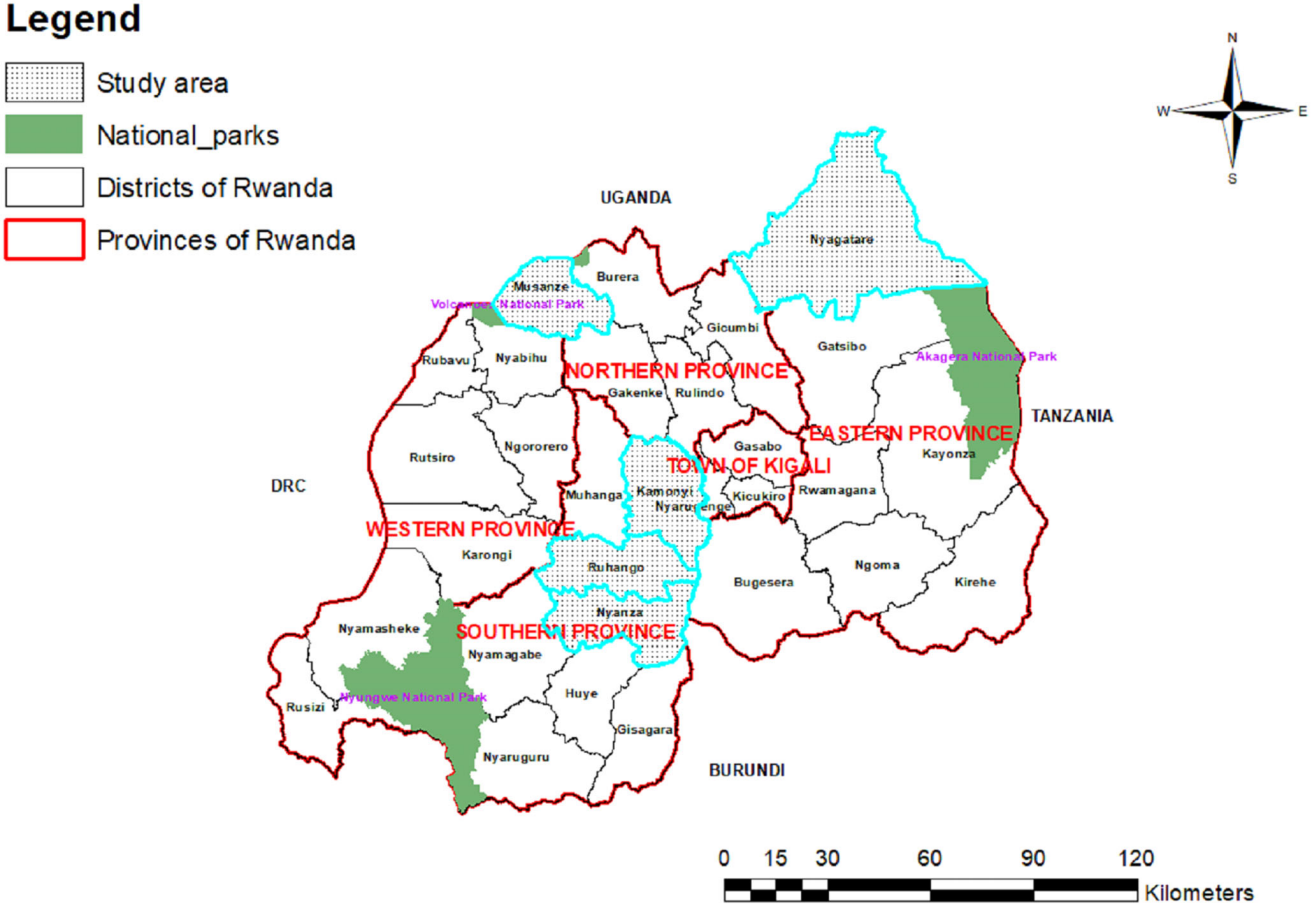
Map of Rwanda with provinces and districts with shaded districts indicating the origin of the samples from aborted animals (map generated in this study)

A cross‐sectional study was carried out from January 2018 through October 2020. The purpose of the study was explained to veterinarians of districts and sectors who were trained on biosafety, and samples of aborted tissues comprising of cotyledons, amniotic fluid, and fetal lungs were collected when available. The study population was cattle that aborted in the district areas (Figures 1 and 2). For cattle, aborted tissues (*n* = 19) comprising of cotyledons and amniotic fluid were collected in the five districts. During the investigation, an abortion storm outbreak occurred in a tribe of goats in the district, and samples were included in this study. Samples of blood (*n* = 10), cotyledons and amniotic fluid (*n* = 1), and vaginal swabs (*n* = 3) were collected from the goats.

### 2.2 Tissue and blood collection

Aborted tissues comprised of cotyledons and amniotic fluids were collected from the aborting cattle into the Falcon 50 ml sterile conical centrifuge tubes (Thermo Fischer Scientific, Johannesburg, South Africa) that were double sealed in biohazard plastic bags by trained veterinarians wearing gloves, masks, and overall. Samples were kept in a cool box containing ice bags and quickly transported to the nearest laboratory for storage at −20°C until further processing. Animals were treated with humane care respecting their welfare. Blood (4 ml) was aseptically collected from the jugular vein of goats into the plain vacutainer tubes by veterinarians. The tubes were transported to the nearest laboratory and incubated overnight at room temperature to allow the serum to separate. The serum was collected into 2 ml Eppendorf tubes and stored at −20°C until further testing.

### 2.3 Serological tests

Goat sera were screened for the presence of anti‐*Brucella* antibodies using the Rose Bengal Test (RBT, IDvet, Grabels, France) according to the manufacturer's instructions. Briefly, sera and *Brucella* antigens were brought to room temperature, and equal volumes were mixed on the wells of the RBT plate using a sterile stick for 4 min. The presence or absence of agglutination was recorded as a positive or negative result, respectively. All sera were subjected to an indirect enzyme‐linked immunosorbent assay (i‐ELISA) according to the manufacturer's instructions (IDvet). The cut‐off value for confirmation of antibody‐positive status in cattle recommended by IDvet is 120%.

### 2.4 *Brucella* isolation from aborted tissues

The tissue samples from cattle and goats were processed and cultured in a certified biosafety level 3 (BSL‐3) facility. Pooled tissues were sliced using sterile scissors and forceps into sterile mortars and grounded using a sterile pestle. An aliquot of pooled homogenate and vaginal swabs were spread into a modified Centro de Investigación y Tecnología Agroalimentaria (CITA) medium and incubated at 37°C with 5.0% CO_2_ atmosphere (Ledwaba et al., 2020). Plates were read for bacterial growth every day for 3 weeks. The morphology of *Brucella* organisms was tested using Stamp's modified Ziehl–Neelsen staining method (OIE, 2021). *Brucella* cultures from modified CITA were subcultured by streaking onto a modified CITA medium to obtain single purified colonies.

### 2.5 Molecular analysis

#### 2.5.1 DNA extraction

Genomic DNA was extracted directly from tissues as well as from cultures using the ReliaPrep gDNA tissue Miniprep system following the manufacturer's guidelines (Promega, Madison, USA). The DNA was kept at −20°C until use.

#### 2.5.2 Detection of the 16S–23S rDNA interspacer sequence (ITS) by PCR

The genus *Brucella* DNA was detected using the 16S–23S rDNA ITS PCR as previously described (Keid et al., 2007). This DNA was screened for the genus *Brucella* spp. using primers (S1) designed from a gene‐specific 16S–23S rDNA interspacer region (ITS), and *B. abortus* RB 51 served as positive controls (Keid et al., 2007). The amplification was done in a thermal cycler (Applied Biosystems 2720, Thermo Fischer Scientific, Foster, USA). The PCR reaction mixture (15 μl) contained 1× of MyTaq Red PCR Mix, primers at 0.2 μM, and 2 μl of template DNA. The PCR cycling condition was initial denaturation at 95°C for 3 min followed by 35 cycles of denaturation at 95°C for 1 min, annealing at 60°C for 2 min, extension at 72°C for 2 min, and a final extension step at 72°C for 5 min. The primers amplified a 214 bp fragment that was analyzed by electrophoresis using a 2% agarose gel stained with red gel nucleic acid stain and visualized under UV light.

#### 2.5.3 Characterization of *Brucella* spp. by AMOS PCR assay

*Brucella abortus* bv. 1, 2, and 4; *B. melitensis* bv. 1, 2, and 3; *B. ovis;* and *B. suis* bv. 1 were identified using a multiplex AMOS PCR assay as previously described (Bricker & Halling, 1994). *Brucella abortus* RB 51 and *B. melitensis* Rev 1 strains DNA served as positive controls. The amplification was performed using a thermal cycler (Applied Biosystems 2720, Thermo Fischer Scientific). A 25 μl reaction mixture contained 1× MyRaq Red PCR Mix, four species‐specific forward primers and reverse primer IS711 (S 1) at a final concentration of 0.1 μM and 0.5 μM, respectively, and 2 μl of template DNA. Thermocycling conditions included initial denaturation at 95°C for 3 min followed by 35 cycles of denaturation at 95°C for 1 min, annealing at 60°C for 2 min, an initial extension at 72°C for 2 min, and a final extension at 72°C for 5 min. PCR products were analyzed by gel electrophoresis using 2% agarose stained with gel red nucleic acid stain and visualized under UV light.

#### 2.5.4 Distinction of *Brucella* spp. from vaccine strains by Bruce‐ladder PCR assay

The identification and distinction of field isolates from vaccine strains of *Brucella* spp. were performed by a multiplex Bruce‐ladder PCR assay as previously described (Garcia‐Yoldi et al., 2006; Lopez‐Goni et al., 2008). *Brucella abortus* bv. 2 REF 544, *B. abortus* RB 51, *B. suis* ZW045 (Ledwaba et al., 2019), and *B. melitensis* Rev 1 strains DNA served as positive controls. The amplification was done in a thermal cycler (Applied Biosystems 2720, Thermo Fischer Scientific). A 25 μl PCR reaction contained 1× MyTaq Red Mix, eight species‐specific forward and reverse primers at a final concentration of 6.25 μM (S 1), and 2 μl of template DNA. The PCR cycling conditions included an initial denaturation at 95°C for 5 min followed by 25 cycles of denaturation at 95°C for 30 s, annealing at 64°C for 45 s, extension at 72°C for 3 min, and a final extension step at 72°C for 10 min. PCR products were analyzed by gel electrophoresis using a 2% agarose stained with gel red nucleic acid stain and viewed under UV light.

#### 2.5.5 Abortion PCR panel

*Brucella* negative samples were screened for *Anaplasma phagocytophilum*, bovine herpes virus type 4, *Campylobacter fetus*, *Chlamydophila* spp., *Coxiella burnetti*, *Leptospira* spp., *Listeria monocytogenes*, and *Salmonella* spp. using a PCR panel.

### 2.6 Data analysis

The proportions of positivity were calculated by dividing the total number of positive animals by the total number of sampled animals. Data were recorded in Microsoft 365 Excel 2013 spreadsheets. Epi‐Info 7 version 10 was used to calculate proportions and determine significant differences between individual risk factors and positive results at 95% confidence intervals using the *χ* ^2^ test. There was a statistical significance if the *p*‐value was < 0.05.

## 3 RESULTS

### 3.1 Detection of *Brucella* spp. in cultures of aborted tissues from cattle

**FIGURE 3:**
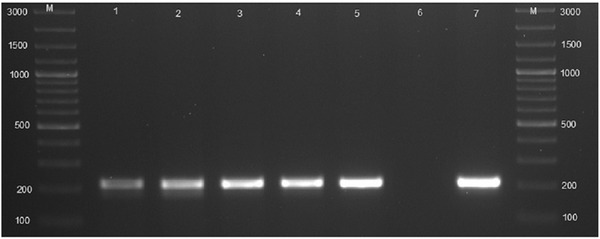
Agarose gel electrophoresis of the 16S–23S ribosomal DNA interspacer region interspacer region (ITS) PCR products amplified from isolates from aborted tissues of cattle and goats. Lanes M: DNA GeneRuler 100 bp (Invitrogen, Thermo Fisher Scientific, Johannesburg, South Africa); lanes 1–2: isolates from aborted tissues of cattle, lanes 3–5: isolates from aborted tissues of goats (amplification of a 214 bp specific *Brucella* DNA region), lane 6: negative control, sterile water; lane 7: positive control, *B. abortus* RB51

**FIGURE 4:**
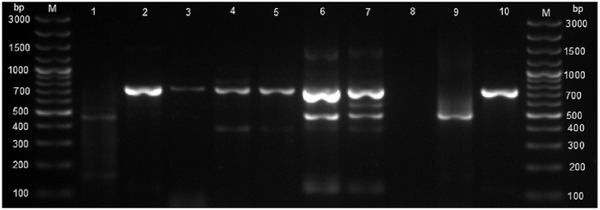
Agarose gel electrophoresis of AMOS PCR products amplified from *Brucella* cultures isolated from tissues of cattle and goats. Lane M: GeneRuler 100 bp (Invitrogen, Thermo Fisher Scientific, Johannesburg, South Africa). Lane 1: *Brucella abortus* 498 bp amplicon from cattle, lane 2: *B. melitensis* 731 bp from cattle, lane 3: *B. melitensis* 731 bp from goats, lanes 6–7 with mixed infection of *B. melitensis* 731 bp and *B. abortus* 498 bp from goat samples, lane 8: negative control sterile water, lane 9: *B. abortus* RB51 strain, lane 10: *B. melitensis* rev 1 strain

**FIGURE 5:**
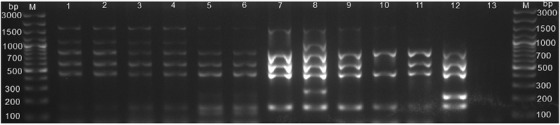
Agarose gel electrophoresis of Bruce‐ladder PCR products amplified from cultures and tissues of cattle and goats. Lane M: GeneRuler 100 bp (Invitrogen, Thermo Fisher Scientific, Johannesburg, South Africa), lanes 1–4: *Brucella melitensis*, lanes 5–7: *B. abortus*, lane 8: *B. suis* ZW 045 strain, lane 9: *B. abortus* bv. 2 REF 544 strain, lane 10: *B. abortus* S19 strain, lane 11: *B. abortus* RB51 strain, lane 12: *B. melitensis* rev 1 strain, lane 13: negative control, sterile water

Out of 19 aborted tissues of cattle, 10.5% (2/19) were from district A, 5.3% (1/19) were from district B, 26.3% (5/19) were district C, 47.4% (9/19) were from district D, and 10.5% (2/19) were from district E. These aborted tissues were cultured onto modified CITA medium, and 84.2% (16/19) had bacterial growth and *Brucella* specific ITS PCR detected 10.5% (2/19) *Brucella* DNA (amplification of a 214 bp sequence, Figure 3). *Brucella melitensis* (*n* = 1) and *B. abortus* (*n* = 1) were detected by AMOS and Bruce‐ladder PCR assays (Figures 4 and 5). In total 10.5% (2/19) culture DNA from aborted tissues of cattle were identified as *Brucella* spp.

### 3.2 Identification of abortigenic pathogens from *Brucella* negative aborted tissues of cattle using PCR panel

*Campylobacter fetus* (*n* = 7) and *Leptospira* spp. (*n* = 4) with two cases of co‐infection caused by *C. fetus* and *Leptospira* spp. were identified from the non‐*Brucella* abortion samples (*n* = 17) using the PCR panel.

### 3.3 Brucellosis of goat's tribe with storm abortion

The abortion storm outbreak occurred in a tribe of 40 dams and three males in June 2019. At the time of the visit, abortion had occurred in 16 dams while the other seven were monitored of which one aborted in our presence. The incidence of abortion was 60.0% (24/40) among the pregnant dams. Sera samples from eight females and two males were RBT and i‐ELISA positive (100.0%, 10/10).

The ITS‐PCR amplified 214 bp *Brucella* DNA for cultures established from the aborted goat tissue (*n* = 1) and vaginal swabs (*n* = 3) (Figure 3). The AMOS PCR detected *B. melitensis* and *B. abortus* with 731 and 498 bp amplification bands, respectively, from *Brucella* cultures isolated from aborted tissue (*n* = 1) and vaginal swabs (*n* = 3) of goats (Figure 4).

## 4 DISCUSSION

This study is the first report of *B. abortus* and *B. melitensis* confirmed with PCR assays from aborted tissues of cattle in the country. This is also the first identification of *C. fetus* and *Leptospira* spp. from aborted tissues of *Brucella* negative samples of cattle using an abortion PCR panel. This study also reports for the first time in the country a mixed infection of *B. abortus* and *B. melitensis* isolated from aborted tissues and vaginal swabs collected during an abortion outbreak of goats in the district D in June 2019. The identified abortigenic pathogens caused considerable financial losses to the animal owners and is a threat to public health.

The causes of abortions include stress (Garcia‐Ispierto & López‐Gatius, 2019; Roth, 2020), nutritional disorders (Akar & Yildiz, 2005), and infectious pathogens such as fungi, viruses, protozoans, and bacteria (Austin, 2021; Barkallah et al., 2014; Leaver & Hart, 1960; Pesca et al., 2020). Abortions due to brucellosis led to the decline of milk production in 1952 with losses amounting to USD 400 million in the United States (Acha & Szyfres, 2003) and negatively affected the livelihood of small farmers in Sub‐Saharan Africa (McDermott et al., 2013). A comparison study of brucellosis seropositive and seronegative pregnant cows in southern Sudan showed that seropositive cows had about 10.0% fewer calves than seronegative cows and abortion occurred in 22.0% of seropositive versus 11.0% of seronegative cows (McDermott et al., 1987). Infectious pathogens contribute to 90% of ruminant abortions and the genus *Brucella* is among the major bacteria that cause abortions in livestock (da Silva et al., 2009; Deresa et al., 2020). This is supported by the isolation of *Brucella* spp. in 10.5% of aborted tissues of cattle in this study.

The absence of *Brucella* spp. in many aborted tissues of cattle led to the screening of *Brucella* negative samples using an abortion PCR panel, which identified *C. fetus* and *Leptospira* spp. This is the first evidence of *C. fetus* and *Leptospira* spp. in aborted tissues of cattle. Infections caused by *Campylobacter* spp. and *Leptospira* spp. have been reported in animals and humans in neighbouring Tanzania (Allan et al., 2020; Gahamanyi et al., 2021) and Uganda (Alinaitwe et al., 2019). This finding calls for active surveillance of genital campylobacteriosis and leptospirosis in aborting cattle, and occupational groups including animal caretakers and abattoir workers.

The combination of AMOS and Bruce‐ladder PCR assays provides cohesive findings because AMOS does not identify all *Brucella* species and biovars but identifies mixed infections, whereas Bruce‐ladder will identify all *Brucella* species and additional biovars but does not detect mixed infections due to the multiple band patterns of Bruce‐ladder PCR assay. With the Bruce‐ladder PCR assay, mixed infections of *B. abortus* and *B. melitensis* will not be detected as *B. melitensis* is identified by 152, 450, 587, 794, 1071, and 1682 bp bands while *B. abortus* is identified by 152, 450, 587, and 794 bp bands and thus the absence of 1071 bp band (Garcia‐Yoldi et al., 2006). The present study identified mixed infections caused by *B. melitensis* and *B. abortus* in goats in Rwanda (Figure 3). *B* *rucella abortus* has been previously reported in goats in Mexico and Egypt (Leal‐Klevezas et al., 2000; Wareth et al., 2015) as well as *B. melitensis* in aborting goats in neighbouring Uganda (Bruce et al., 1910; Philpott & Auko, 1972), Tanzania (Philpott & Auko, 1972), and Kenya (Muendo et al., 2012; Philpott & Auko, 1972). The mixed infections by *B. melitensis* and *B. abortus* in all the four abortion samples of goats indicate the cross‐infection and inappropriate management of herding different animal species on the same farm (Ocholi et al., 2005). Goats of the present study shared the same grazing pasture with cattle indicating shedding of aborted tissues in the pasture and the high risk of interspecies transmission. In the present study, males of the tribe were also seropositive to brucellosis, and this contributed to the propagation of disease in the whole tribe.

The introduction of caprine brucellosis in the country may be associated with uncontrolled repatriation of citizens and their livestock, or importation of chronically diseased goats for the distribution to poor families. Although livestock are screened for brucellosis before importation, animals in early incubation, or chronically diseased may be seronegative due to the decline of antibody titres but remain bacteriologically positive (Morgan & McDiarmid, 1960; Nicoletti & Muraschi, 1966; Zowghi et al., 1990). Therefore, screening before importation followed by quarantine and second serological screening would guarantee the brucellosis‐free status.

Caprine brucellosis constitutes a public health concern because *B. melitensis* causes severe disease in humans (Bruce, 1887; Wallach et al., 1997). A study has demonstrated a significant association between caprine brucellosis and brucellosis in owners of goats (Miller et al., 2016). It was thought for a long time that there is no brucellosis in goats in the country, and the vaccination programme against brucellosis targets exclusively cattle. The discovery of *B. melitensis* and *B. abortus* in goats has public health implications since there exist few households still sleeping in the same house with their goats to prevent stealing, and this may favour inhalation of *Brucella* spp. aerosols if ventilation is not sufficient in the houses (Kaufmann et al., 1980). In addition, there was a significant association between caprine and bovine brucellosis; and goats play an important role in the transmission of brucellosis to cattle (Miller et al., 2016).

This study isolated *B. melitensis* and *B. abortus* in aborted tissues of cattle. A similar study in India isolated *B. abortus* and *B. melitensis* from different reproductive tissues of buffaloes, cows, does, and ewes (Verma et al., 2000). The proportion of isolation of *Brucella* spp. from aborted tissues of cattle (10.5%) obtained in the present study is comparable with serosurvey studies that reported abortions in 16.2% of seropositive cattle in Zambia (Muma et al., 2007) and 13.8% found in Ethiopia (Megersa et al., 2011).

The abortion cases reported in the present study caused tremendous financial losses in the livestock industry in the country. The vaccination against brucellosis that currently focuses only on cattle should be expanded to include goats and sheep, preferably in a systematic and coordinated manner. The control programme against brucellosis should focus on the hygiene of the animal environment, provision of separate maternity pen, early weaning, and before introduction into the herd or flock; animals should be screened using both buffered agglutination test like RBT and a confirmation test either ELISA or complement fixation test to distinguish early and latent infections. Any abortion case should be reported to the competent authority and the herd or tribe should be massively screened against brucellosis, and the positive animals should be immediately slaughtered to stop spreading. *Brucella* negative animals should be screened for other abortigenic pathogens such as *C. fetus*, *Leptospira* spp. that were detected in this study (See Supporting Information).

## 5 CONCLUSIONS

This study identified for the first time *B. melitensis*, *B. abortus*, *C. fetus*, and *Leptospira* spp. from aborted tissues of cattle. Mixed infections caused by *C. fetus* and *Leptospira* spp. were recorded in cattle indicating the severity of abortion in the herd. Co‐infections of *B. melitensis* and *B. abortus* in aborted tissues of goats indicated cross‐infection in cattle and goats. These abortions caused tremendous financial losses in the livestock industry in the country. Any abortion case should be reported to the competent authority and the herd or flock should be massively screened against brucellosis. Consecutively, positive animals should be slaughtered immediately to stop spreading. It is also important to screen all *Brucella* seronegative animals for other abortigenic pathogens to control ruminant abortions in the country.

## CONFLICT OF INTEREST

The authors declare no conflict of interest.

## ETHICS STATEMENT

The authors confirm that the ethical policies of the journal, as noted on the journal's author guidelines page, have been adhered to and the appropriate ethical review committee approval has been received. The US National Research Council's guidelines for the Care and Use of Laboratory Animals were followed.

## AUTHOR CONTRIBUTIONS

*Conceptualization*: Jean Bosco Ntivuguruzwa and Henriette van Heerden. *Methodology*: Jean Bosco Ntivuguruzwa and Henriette van Heerden. *Formal analysis*: Jean Bosco Ntivuguruzwa. *Investigation and data collection*: Jean Bosco Ntivuguruzwa. *Writing—original draft preparation*: Jean Bosco Ntivuguruzwa. *Writing—review and editing*: Jean Bosco Ntivuguruzwa, Emil Ivan Mwikarago: Francis Babaman Kolo and Henriette van Heerden. *Supervision*: Henriette van Heerde and Francis Babaman Kolo. *Project administration*: Henriette van Heerden. *Resources*: Henriette van Heerden. *Funding acquisition and Validation*: Henriette van Heerden.

### PEER REVIEW

The peer review history for this article is available at https://publons.com/publon/10.1002/vms3.805.

## Supporting information



Supporting Information 1

Supporting Information 1

Supporting Information 1

## Data Availability

The data that supports the findings of this study are available in the supplementary material of this article.
