# Supplementary material for: Characterization of Brucella spp. and other abortigenic pathogens from aborted tissues of cattle and goats in Rwanda
Source: Vet Med Sci. 2022 Apr 14;8(4):1655–63. doi: 10.1002/vms3.805 (PMC9297783; doi:10.1002/vms3.805)
Supplement: Supplementary file 1 — Supporting Information 1 [file VMS3-8-1655-s002.docx]

S1. Sequences of oligonucleotide primers used for the detection and distinction of *Brucella* spp. using the 16S-23S ribosomal DNA interspacer region (ITS), AMOS, and Bruce-ladder PCR assays.

| **PCR name** | **Primer name** | **Sequence (5'-3')** | **Targets** | **Size (bp)** | **Conc. (µM)** | **References** |
| --- | --- | --- | --- | --- | --- | --- |
| ITS | ITS66 f | ACATAGATCGCAGGCCAGTCA | *16s-23s rRNA* | 214 | 0.2 | (Keid et al., 2007) |
|  | ITS279r | ACATAGATCGCAGGCCAGTCA |  |  |  |  |
| A | *B. abortus* | GAC GAA CGG AAT TTT TCC AAT CCC | *IS711* | 498 | 0.1 | (Bricker & Halling, 1994) |
| M | *B. melitensis* | AAA TCG CGT CCT TGC TGG TCT GA |  | 731 | 0.1 |  |
| O | *B. ovis* | CGG GTT CTG GCA CCA TCG TCG GG |  | 976 | 0.1 |  |
| S | *B. suis* | GCG CGG TTT TCT GAA GGT GGT TCA |  | 285 | 0.1 |  |
|  | *IS 711* | TGC CGA TCA CTT AAG GGC CTT CAT |  | - | 0.2 |  |
| BRUCE- LADDER | BMEI0998f | ATC CTA TTG CCC CGA TAA GG | *wboA* | 1682 | 6.25 | (Garcia-Yoldi, Marín, & Lopez-Goni, 2005; Vemulapalli et al., 1999) |
|  | BMEI0997r | GCT TCG CAT TTT CAC TGT AGC |  |  |  |  |
|  | BMEI0535f | GCG CAT TCT TCG GTT ATG AA | *bp26* | 450 | 6.25 | (Cloeckaert, Grayon, & Grepinet, 2000) |
|  | BMEI0536r | CGC AGG CGA AAA CAG CTA TAA |  |  |  |  |
|  | BMEII0843f | TTT ACA CAG GCA ATC CAG CA | *omp31* | 1071 | 6.25 | (Vizcaino, Verger, Grayon, Zygmunt, & Cloeckaert, 1997) |
|  | BMEII0844r | GCG TCC AGT TGT TGT TGA TG |  |  |  |  |
|  | BMEI1436f | ACG CAG ACG ACC TTC GGT AT | *Deacetylase* | 794 | 6.25 | (Rajashekara, Glasner, Glover, & Splitter, 2004) |
|  | BMEI1435r | TTT ATC CAT CGC CCT GTC AC |  |  |  |  |
|  | BMEII0428f | GCC GCT ATT ATG TGG ACT GG | *eryC* | 587 | 6.25 | (Sangari, García-Lobo, & Agüero, 1994) |
|  | BMEII0428r | AAT GAC TTC ACG GTC GTTCG |  |  |  |  |
|  | BR0953f | GGA ACA CTA CGC CAC CTT GT | *ABC Transporter* | 272 | 6.25 | (Halling et al., 2005) |
|  | BR0953r | GAT GGA GCA AAC GCT GAA G |  |  |  |  |
|  | BMEI0752f | CAG GCA AAC CCT CAG AAG C | *rpsL* | 218 | 6.25 | (Cloeckaert, Grayon, & Grépinet, 2002) |
|  | BMEI0752r | GAT GTG GTA ACG CAC ACC AA |  |  |  |  |
|  | BMEII0987f | CGC AGA CAG TGA CCA TCA AA | *CRP Regulator* | 152 | 6.25 | (Rajashekara et al., 2004) |
|  | BMEII0987r | GTA TTC AGC CCC CGT TAC CT |  |  |  |  |

CLOECKAERT, A., GRAYON, M., & GREPINET, O. 2000. An IS711 element downstream of the bp26 gene is a specific marker of *Brucella spp.* isolated from marine mammals. *Clinical and Diagnostic Laboratory Immunology, 7*(5), 835-839.

CLOECKAERT, A., GRAYON, M., & GRÉPINET, O. 2002. Identification of *Brucella melitensis* vaccine strain Rev.1 by PCR-RFLP based on a mutation in the rpsL gene. *Vaccine, 20*(19), 2546-2550. doi:<https://doi.org/10.1016/S0264-410X(02)00159-7>

GARCIA-YOLDI, D., MARÍN, C., & LOPEZ-GONI, I. 2005. Restriction site polymorphisms in the genes encoding new members of group 3 outer membrane protein family of Brucella spp. *FEMS Microbiology Letters, 245*(1), 79-84.

HALLING, S. M., PETERSON-BURCH, B. D., BRICKER, B. J., ZUERNER, R. L., QING, Z., LI, L.-L., . . . OLSEN, S. C. 2005. Completion of the genome sequence of *Brucella abortus* and comparison to the highly similar genomes of *Brucella melitensis* and *Brucella suis*. *Journal of Bacteriology, 187*(8), 2715-2726.

RAJASHEKARA, G., GLASNER, J. D., GLOVER, D. A., & SPLITTER, G. A. 2004. Comparative whole-genome hybridization reveals genomic islands in *Brucella* species. *Journal of Bacteriology, 186*(15), 5040-5051.

SANGARI, F. J., GARCIA-LOBO, J. M., & AGÜERO, J. 1994. The *Brucella abortus* vaccine strain B19 carries a deletion in the erythritol catabolic genes. *FEMS Microbiology Letters, 121*(3), 337-342.

VEMULAPALLI, R., MCQUISTON, J. R., SCHURIG, G. G., SRIRANGANATHAN, N., HALLING, S. M., & BOYLE, S. M. 1999. Identification of an IS711 Element Interrupting the *wboA* Gene of *Brucella abortus* Vaccine Strain RB51 and a PCR Assay To Distinguish Strain RB51 from Other *Brucella* Species and Strains. *Clinical and Diagnostic Laboratory Immunology, 6*(5), 760-764.

VIZCAINO, N., VERGER, J.-M., GRAYON, M., ZYGMUNT, M. S., & CLOECKAERT, A. 1997. DNA polymorphism at the omp-31 locus of *Brucella* spp.: evidence for a large deletion in *Brucella abortus*, and other species-specific markers. *Microbiology, 143*(9), 2913-2921.WALLACH, J. C., SAMARTINO, L. E., EFRON, A. & BALDI, P. C. 1997. Human infection by *Brucella melitensis*: an outbreak attributed to contact with infected goats. *FEMS Immunology & Medical Microbiology,* 19**,** 315-321.
