# Supplementary material for: Characterization of Brucella spp. and other abortigenic pathogens from aborted tissues of cattle and goats in Rwanda
Source: Vet Med Sci. 2022 Apr 14;8(4):1655–63. doi: 10.1002/vms3.805 (PMC9297783; doi:10.1002/vms3.805)
Supplement: Supplementary file 3 — Supporting Information 1 [file VMS3-8-1655-s003.pdf]

MOLECULAR DIAGNOSTIC SERVICES (PTY) LTD

6 Ribston Place, Westville, 3629 • Pvt Bag X20, Westville 3630, South Africa
Tel: +27 31 267 7000 • Fax: +27 31 267 7005 • Email: reception@mdsafrica.net
Vat Reg No: 427 0210554 • SAVC No: ART 06/7930
Registration Number: 2001/001 77907
www.mdsafrica.net

VETERINARY MOLECULAR RESULT - FINAL REPORT

CLIENT: University of Pretoria, Faculty of Veterinary Science
Facalty of Vet
TEL: 012 529 8161/0125298073
CELL: 0125298334
FAX:
EMAIL: johan.steyl@up.ac.za
ADDRESS: Private bag x 04
Onderstepoort
Pretoria
0110

RN#: RN051516
DATE RECEIVED: 04 Mar 2021
DATE AUTHORISED: 25 Mar 2021
DATE SAMPLE/S COLLECTED: 02 Mar 2021
CLIENT REFERENCE: Test samples individually - samples from 2019

Date Tested	MDS No.	*Owner	*Species	*Animal ID	*Sample Type	*Test	Result	Comment
19 Mar 2021	513488	Unknown - Farmer from Rwanda	Bovine	Ab 01	Extracted DNA	Anaplasma phagocytophilum	*	[1]
19 Mar 2021	513488	Unknown - Farmer from Rwanda	Bovine	Ab 01	Extracted DNA	Bovine Herpes Virus Type 4	*	[1]
19 Mar 2021	513488	Unknown - Farmer from Rwanda	Bovine	Ab 01	Extracted DNA	Campylobacter fetus	*	[1]
19 Mar 2021	513488	Unknown - Farmer from Rwanda	Bovine	Ab 01	Extracted DNA	Chlamydophila spp	*	[1]
19 Mar 2021	513488	Unknown - Farmer from Rwanda	Bovine	Ab 01	Extracted DNA	Coxiella burnetii	*	[1]
19 Mar 2021	513488	Unknown - Farmer from Rwanda	Bovine	Ab 01	Extracted DNA	Leptospira pathogenes	*	[1]
19 Mar 2021	513488	Unknown - Farmer from Rwanda	Bovine	Ab 01	Extracted DNA	Listeria monocytogenes	*	[1]
19 Mar 2021	513488	Unknown - Farmer from Rwanda	Bovine	Ab 01	Extracted DNA	Salmonella spp.	*	[1]
19 Mar 2021	513489	Unknown - Farmer from Rwanda	Bovine	Ab 02	Extracted DNA	Anaplasma phagocytophilum	Not Detected	
19 Mar 2021	513489	Unknown - Farmer from Rwanda	Bovine	Ab 02	Extracted DNA	Bovine Herpes Virus Type 4	Not Detected	
19 Mar 2021	513489	Unknown - Farmer from Rwanda	Bovine	Ab 02	Extracted DNA	Campylobacter fetus	Positive	
19 Mar 2021	513489	Unknown - Farmer from Rwanda	Bovine	Ab 02	Extracted DNA	Chlamydophila spp	Not Detected	
19 Mar 2021	513489	Unknown - Farmer from Rwanda	Bovine	Ab 02	Extracted DNA	Coxiella burnetii	Not Detected	

19 Mar 2021	513489	Unknown - Farmer from Rwanda	Bovine	Ab 02	Extracted DNA	Leptospira pathogenes	Not Detected	
19 Mar 2021	513489	Unknown - Farmer from Rwanda	Bovine	Ab 02	Extracted DNA	Listeria monocytogenes	Not Detected	
19 Mar 2021	513489	Unknown - Farmer from Rwanda	Bovine	Ab 02	Extracted DNA	Salmonella spp.	Not Detected	
19 Mar 2021	513490	Unknown - Farmer from Rwanda	Bovine	Ab 03	Extracted DNA	Anaplasma phagocytophilum	Not Detected	
19 Mar 2021	513490	Unknown - Farmer from Rwanda	Bovine	Ab 03	Extracted DNA	Bovine Herpes Virus Type 4	Not Detected	
19 Mar 2021	513490	Unknown - Farmer from Rwanda	Bovine	Ab 03	Extracted DNA	Campylobacter fetus	Not Detected	
19 Mar 2021	513490	Unknown - Farmer from Rwanda	Bovine	Ab 03	Extracted DNA	Chlamydophila spp	Not Detected	
19 Mar 2021	513490	Unknown - Farmer from Rwanda	Bovine	Ab 03	Extracted DNA	Coxiella burnetii	Not Detected	
19 Mar 2021	513490	Unknown - Farmer from Rwanda	Bovine	Ab 03	Extracted DNA	Leptospira pathogenes	Not Detected	
19 Mar 2021	513490	Unknown - Farmer from Rwanda	Bovine	Ab 03	Extracted DNA	Listeria monocytogenes	Not Detected	
19 Mar 2021	513490	Unknown - Farmer from Rwanda	Bovine	Ab 03	Extracted DNA	Salmonella spp.	Not Detected	
25 Mar 2021	513491	Unknown - Farmer from Rwanda	Bovine	Ab 04	Extracted DNA	Campylobacter fetus	Not Detected	
25 Mar 2021	513491	Unknown - Farmer from Rwanda	Bovine	Ab 04	Extracted DNA	Leptospira spp.	Positive	
25 Mar 2021	513492	Unknown - Farmer from Rwanda	Bovine	Ab 05	Extracted DNA	Campylobacter fetus	Not Detected	
25 Mar 2021	513492	Unknown - Farmer from Rwanda	Bovine	Ab 05	Extracted DNA	Leptospira spp.	Not Detected	
25 Mar 2021	513493	Unknown - Farmer from Rwanda	Bovine	Ab 07	Extracted DNA	Campylobacter fetus	Not Detected	
25 Mar 2021	513493	Unknown - Farmer from Rwanda	Bovine	Ab 07	Extracted DNA	Leptospira spp.	Not Detected	
25 Mar 2021	513494	Unknown - Farmer from Rwanda	Bovine	Ab 09	Extracted DNA	Campylobacter fetus	Not Detected	
25 Mar 2021	513494	Unknown - Farmer from Rwanda	Bovine	Ab 09	Extracted DNA	Leptospira spp.	Not Detected	
19 Mar 2021	513495	Unknown - Farmer from Rwanda	Bovine	Ab 13	Extracted DNA	Anaplasma phagocytophilum	Not Detected	
19 Mar 2021	513495	Unknown - Farmer from Rwanda	Bovine	Ab 13	Extracted DNA	Bovine Herpes Virus Type 4	Not Detected	

19 Mar 2021	513495	Unknown - Farmer from Rwanda	Bovine	Ab 13	Extracted DNA	Campylobacter fetus	Positive	
19 Mar 2021	513495	Unknown - Farmer from Rwanda	Bovine	Ab 13	Extracted DNA	Chlamydophila spp	Not Detected	
19 Mar 2021	513495	Unknown - Farmer from Rwanda	Bovine	Ab 13	Extracted DNA	Coxiella burnetii	Not Detected	
19 Mar 2021	513495	Unknown - Farmer from Rwanda	Bovine	Ab 13	Extracted DNA	Leptospira pathogenes	Not Detected	
19 Mar 2021	513495	Unknown - Farmer from Rwanda	Bovine	Ab 13	Extracted DNA	Listeria monocytogenes	Not Detected	
19 Mar 2021	513495	Unknown - Farmer from Rwanda	Bovine	Ab 13	Extracted DNA	Salmonella spp.	Not Detected	
19 Mar 2021	513496	Unknown - Farmer from Rwanda	Bovine	Ab 14	Extracted DNA	Anaplasma phagocytophilum	Not Detected	
19 Mar 2021	513496	Unknown - Farmer from Rwanda	Bovine	Ab 14	Extracted DNA	Bovine Herpes Virus Type 4	Not Detected	
19 Mar 2021	513496	Unknown - Farmer from Rwanda	Bovine	Ab 14	Extracted DNA	Campylobacter fetus	Positive	
19 Mar 2021	513496	Unknown - Farmer from Rwanda	Bovine	Ab 14	Extracted DNA	Chlamydophila spp	Not Detected	
19 Mar 2021	513496	Unknown - Farmer from Rwanda	Bovine	Ab 14	Extracted DNA	Coxiella burnetii	Not Detected	
19 Mar 2021	513496	Unknown - Farmer from Rwanda	Bovine	Ab 14	Extracted DNA	Leptospira pathogenes	Not Detected	
19 Mar 2021	513496	Unknown - Farmer from Rwanda	Bovine	Ab 14	Extracted DNA	Listeria monocytogenes	Not Detected	
19 Mar 2021	513496	Unknown - Farmer from Rwanda	Bovine	Ab 14	Extracted DNA	Salmonella spp.	Not Detected	
19 Mar 2021	513497	Unknown - Farmer from Rwanda	Bovine	Ab 15	Extracted DNA	Anaplasma phagocytophilum	Not Detected	
19 Mar 2021	513497	Unknown - Farmer from Rwanda	Bovine	Ab 15	Extracted DNA	Bovine Herpes Virus Type 4	Not Detected	
19 Mar 2021	513497	Unknown - Farmer from Rwanda	Bovine	Ab 15	Extracted DNA	Campylobacter fetus	Positive	
19 Mar 2021	513497	Unknown - Farmer from Rwanda	Bovine	Ab 15	Extracted DNA	Chlamydophila spp	Not Detected	
19 Mar 2021	513497	Unknown - Farmer from Rwanda	Bovine	Ab 15	Extracted DNA	Coxiella burnetii	Not Detected	
19 Mar 2021	513497	Unknown - Farmer from Rwanda	Bovine	Ab 15	Extracted DNA	Leptospira pathogenes	Positive	
19 Mar 2021	513497	Unknown - Farmer from Rwanda	Bovine	Ab 15	Extracted DNA	Listeria monocytogenes	Not Detected	

19 Mar 2021	513497	Unknown - Farmer from Rwanda	Bovine	Ab 15	Extracted DNA	Salmonella spp.	Not Detected	
19 Mar 2021	513498	Unknown - Farmer from Rwanda	Bovine	Ab 16	Extracted DNA	Anaplasma phagocytophilum	Not Detected	
19 Mar 2021	513498	Unknown - Farmer from Rwanda	Bovine	Ab 16	Extracted DNA	Bovine Herpes Virus Type 4	Not Detected	
19 Mar 2021	513498	Unknown - Farmer from Rwanda	Bovine	Ab 16	Extracted DNA	Campylobacter fetus	Positive	
19 Mar 2021	513498	Unknown - Farmer from Rwanda	Bovine	Ab 16	Extracted DNA	Chlamydophila spp	Not Detected	
19 Mar 2021	513498	Unknown - Farmer from Rwanda	Bovine	Ab 16	Extracted DNA	Coxiella burnetii	Not Detected	
19 Mar 2021	513498	Unknown - Farmer from Rwanda	Bovine	Ab 16	Extracted DNA	Leptospira pathogenes	Not Detected	
19 Mar 2021	513498	Unknown - Farmer from Rwanda	Bovine	Ab 16	Extracted DNA	Listeria monocytogenes	Not Detected	
19 Mar 2021	513498	Unknown - Farmer from Rwanda	Bovine	Ab 16	Extracted DNA	Salmonella spp.	Not Detected	
25 Mar 2021	513499	Unknown - Farmer from Rwanda	Bovine	Ab 17	Extracted DNA	Campylobacter fetus	Weak Positive	
25 Mar 2021	513499	Unknown - Farmer from Rwanda	Bovine	Ab 17	Extracted DNA	Leptospira spp.	Positive	
19 Mar 2021	513500	Unknown - Farmer from Rwanda	Bovine	Ab 18	Extracted DNA	Anaplasma phagocytophilum	Not Detected	
19 Mar 2021	513500	Unknown - Farmer from Rwanda	Bovine	Ab 18	Extracted DNA	Bovine Herpes Virus Type 4	Not Detected	
19 Mar 2021	513500	Unknown - Farmer from Rwanda	Bovine	Ab 18	Extracted DNA	Campylobacter fetus	Positive	
19 Mar 2021	513500	Unknown - Farmer from Rwanda	Bovine	Ab 18	Extracted DNA	Chlamydophila spp	Not Detected	
19 Mar 2021	513500	Unknown - Farmer from Rwanda	Bovine	Ab 18	Extracted DNA	Coxiella burnetii	Not Detected	
19 Mar 2021	513500	Unknown - Farmer from Rwanda	Bovine	Ab 18	Extracted DNA	Leptospira pathogenes	Not Detected	
19 Mar 2021	513500	Unknown - Farmer from Rwanda	Bovine	Ab 18	Extracted DNA	Listeria monocytogenes	Not Detected	
19 Mar 2021	513500	Unknown - Farmer from Rwanda	Bovine	Ab 18	Extracted DNA	Salmonella spp.	Not Detected	
19 Mar 2021	513501	Unknown - Farmer from Rwanda	Bovine	Ab 20	Extracted DNA	Anaplasma phagocytophilum	Not Detected	
19 Mar 2021	513501	Unknown - Farmer from Rwanda	Bovine	Ab 20	Extracted DNA	Bovine Herpes Virus Type 4	Not Detected	

19 Mar 2021	513501	Unknown - Farmer from Rwanda	Bovine	Ab 20	Extracted DNA	Campylobacter fetus	Not Detected	
19 Mar 2021	513501	Unknown - Farmer from Rwanda	Bovine	Ab 20	Extracted DNA	Chlamydophila spp	Not Detected	
19 Mar 2021	513501	Unknown - Farmer from Rwanda	Bovine	Ab 20	Extracted DNA	Coxiella burnetii	Not Detected	
19 Mar 2021	513501	Unknown - Farmer from Rwanda	Bovine	Ab 20	Extracted DNA	Leptospira pathogenes	Not Detected	
19 Mar 2021	513501	Unknown - Farmer from Rwanda	Bovine	Ab 20	Extracted DNA	Listeria monocytogenes	Not Detected	
19 Mar 2021	513501	Unknown - Farmer from Rwanda	Bovine	Ab 20	Extracted DNA	Salmonella spp.	Not Detected	
19 Mar 2021	513502	Unknown - Farmer from Rwanda	Bovine	Ab/BC	Extracted DNA	Anaplasma phagocytophilum	Not Detected	
19 Mar 2021	513502	Unknown - Farmer from Rwanda	Bovine	Ab/BC	Extracted DNA	Bovine Herpes Virus Type 4	Not Detected	
19 Mar 2021	513502	Unknown - Farmer from Rwanda	Bovine	Ab/BC	Extracted DNA	Campylobacter fetus	Not Detected	
19 Mar 2021	513502	Unknown - Farmer from Rwanda	Bovine	Ab/BC	Extracted DNA	Chlamydophila spp	Not Detected	
19 Mar 2021	513502	Unknown - Farmer from Rwanda	Bovine	Ab/BC	Extracted DNA	Coxiella burnetii	Not Detected	
19 Mar 2021	513502	Unknown - Farmer from Rwanda	Bovine	Ab/BC	Extracted DNA	Leptospira pathogenes	Not Detected	
19 Mar 2021	513502	Unknown - Farmer from Rwanda	Bovine	Ab/BC	Extracted DNA	Listeria monocytogenes	Not Detected	
19 Mar 2021	513502	Unknown - Farmer from Rwanda	Bovine	Ab/BC	Extracted DNA	Salmonella spp.	Not Detected	
19 Mar 2021	513503	Unknown - Farmer from Rwanda	Bovine	4b	Extracted DNA	Anaplasma phagocytophilum	Not Detected	
19 Mar 2021	513503	Unknown - Farmer from Rwanda	Bovine	4b	Extracted DNA	Bovine Herpes Virus Type 4	Not Detected	
19 Mar 2021	513503	Unknown - Farmer from Rwanda	Bovine	4b	Extracted DNA	Campylobacter fetus	Not Detected	
19 Mar 2021	513503	Unknown - Farmer from Rwanda	Bovine	4b	Extracted DNA	Chlamydophila spp	Not Detected	
19 Mar 2021	513503	Unknown - Farmer from Rwanda	Bovine	4b	Extracted DNA	Coxiella burnetii	Not Detected	
19 Mar 2021	513503	Unknown - Farmer from Rwanda	Bovine	4b	Extracted DNA	Leptospira pathogenes	Not Detected	
19 Mar 2021	513503	Unknown - Farmer from Rwanda	Bovine	4b	Extracted DNA	Listeria monocytogenes	Not Detected	

19 Mar 2021	513503	Unknown - Farmer from Rwanda	Bovine	4b	Extracted DNA	Salmonella spp.	Not Detected	
25 Mar 2021	513504	Unknown - Farmer from Rwanda	Bovine	Abc 2020	Extracted DNA	Campylobacter fetus	Not Detected	
25 Mar 2021	513504	Unknown - Farmer from Rwanda	Bovine	Abc 2020	Extracted DNA	Leptospira spp.	Query positive	
25 Mar 2021	513505	Unknown - Farmer from Rwanda	Bovine	Ab 19	Extracted DNA	Campylobacter fetus	Not Detected	
25 Mar 2021	513505	Unknown - Farmer from Rwanda	Bovine	Ab 19	Extracted DNA	Leptospira spp.	Not Detected	

Sample Condition: Satisfactory unless otherwise indicated; refer to individual sample comment.

INDIVIDUAL RESULT COMMENT/S:

[1] Please note: no housekeeping gene internal control was detected in this sample. We are therefore unable to determine if there were any inhibitors present in the extract. Please submit a primary sample if confirmatory testing is required.

REPORT AUTHORISED BY: Maireschka Smith (D12/9290)

General Comment:
The reported test result/s relate only to the specific sample/s submitted and the status of the bird / animal at the time that the specific sample was collected and as received by the testing laboratory. The information in the columns marked with an asterisk (*) is provided by the customer. MDS does not accept responsibility for incorrect information submitted by the client, that may affect validity of the result. These results were generated by MDS using nucleic acid amplification procedures. Each test done by MDS is internally controlled to validate the testing process and the collected sample.

MDS is not responsible for sampling or environmental conditions that may affect the validity of the result prior to the specific sample arriving at the testing laboratory.

Disclaimer:
In the event that Molecular Diagnostic Services (Pty) Ltd (MDS) returns a verifiable erroneous result for a particular sample, or if negligence is proven on the part of MDS, any claim against MDS is limited to the amount paid or to be paid to perform the test in question. In no event shall MDS be liable for direct, indirect, incidental, consequential, special or other damages of any nature even if MDS has been advised of the possibility of such damage. Should any details on this form be incorrect then they must be brought to the attention of MDS. Any alteration or modification of the information on this form is not authorised and legal action will be taken against any person found guilty of such action. We realize the importance of confidentiality in the service we provide and undertake to abide by our Privacy Policy. For further information about our Privacy Policy, please contact our office or visit our website www.mdsafrica.net.

FOR RESULT QUERIES – Please call 031 267 7000 or email vetsite@mdsafrica.net
Director: Dr DF York; Lab Manager: Ms L-A Edwards; Veterinary Department; Bioinformatics Department (sequencing queries)

END OF REPORT
